# Single-shot probing of sub-picosecond solid-to-overdense-plasma dynamics

**DOI:** 10.1038/s41377-024-01501-6

**Published:** 2024-07-11

**Authors:** Kunjian Dai, Qingzhe Cui, Jinwei Zhang

**Affiliations:** School of Optical and Electronic Information and Wuhan National Laboratory for Optoelectronics, Huazhong University of Science and Technology, Wuhan, 430074 China

**Keywords:** Laser-produced plasmas, Ultrafast photonics

## Abstract

A single-shot near-infrared probing method has been developed to characterize the formation and evolution of the pre-plasma dynamics over sub-picosecond timescales, which is essential for the societal applications of laser-accelerated ion technologies.

When irradiated by intense ultrashort laser pulses, a solid target will be ionized, resulting in the creation of high-temperature and overdense plasma. Plasma, a state of matter consisting of positively charged ions and negatively charged electrons, is usually utilized to generate high-energy particles under the influence of intense laser fields^1,2^. Characterizing plasma dynamics is essential, as the evolution of plasmas directly impacts the efficiency of the laser-matter interaction^3,4^ and the properties of the subsequently generated high-energy particles^5,6^. However, the complex nature of the plasma dynamics emphasizes the significance of precise measurement. Hence, accurate simulation modeling^7^ and experimental measurement^8,9^ of the plasma generation process are imperative for optimizing the controls over laser pulse parameters, target properties, and interaction dynamics which are of great importance for improving the quality of applications related to high-energy particles^10,11^. For conventional modeling approaches using particle-in-cell (PIC) simulations^12^, the pre-plasma dynamics are always ignored, while from experimental perspectives, a single-shot measurement of the pre-plasma evolution is challenging.

In a recently published paper in *Light: Science & Applications*, Azamoum et al.^13^ demonstrated the experimental observation of pre-plasma dynamics over sub-picosecond timescales through single-shot near-infrared probe transmission measurements. By irradiating nm-thin diamond-like carbon (DLC) foils with femtosecond laser pulses with intensities exceeding 10^16^ W/cm^2^, as illustrated in Fig. 1a, the authors observed the formation of pre-plasma within a specific time window which describes the steep rising edge of the ultrashort pulse. The pre-plasma dynamics were diagnosed using a temporally chirped broadband probe pulse, where the wavelength information was converted into time to measure the ultrafast transition. The penetration of the probing light pulse, which is related to how light propagates in plasma, was used to analyze the pre-plasma dynamics. It turns out that when the electron density *n*_*e*_ is larger than the critical density *n*_*c*_, a fraction of the probe light can propagate through the plasma over the skin depth, ${l}_{s}\approx c/{\omega }_{p}$, where *ω*_*p*_ is the plasma frequency. The probe light tunnels through when the generated pre-plasma is thinner than *l*_*s*_ which works as an investigation of the pre-plasma dynamics from solid-to-overdense-plasma. The probe transmission map was captured with a 1D spatially resolved imaging spectrometer, as shown in Fig. 1c. The map shows that wavelength components smaller than 820 nm in the probe are blocked due to the pre-plasma transition. This optical probing approach enables the investigation of plasma dynamics in single-shot measurements. In order to explain the experimental findings, the authors developed a two-step interaction (TSI) simulation model, which combines the solid-state interaction (SSI) model^14^ and the PIC model. In the TSI simulation, the authors calculated the ionization of the solid target with SSI model on the initial stage of the pre-plasma dynamics, and then used the PIC model to describe the expansion of the plasma. The measured and calculated transmission for the DLC foil with a thickness of 20 nm is shown in Fig. 1b. It can be seen that the experimentally observed transition was accurately described by the TSI model.

**Fig. 1:**
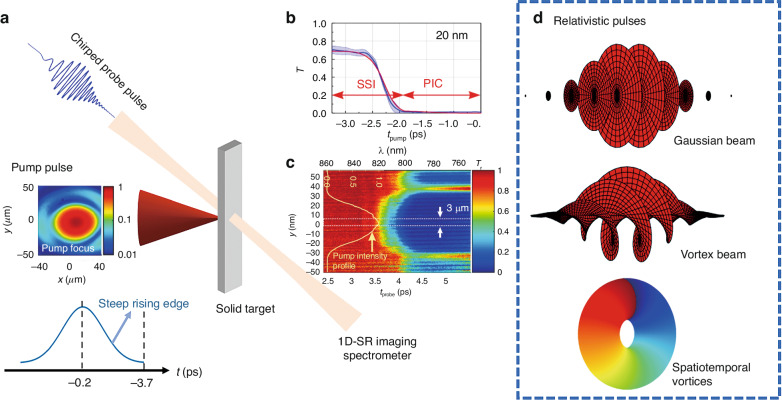
Schematic view of single-shot probing of the ultrafast laser-induced solid-to-overdense-plasma transitions. **a** The experimental arrangement of the single-shot near-infrared probe measurement. **b** The measured and calculated transmission for a foil with a thickness of 20 nm. **c** The probe transmission map measured with a 1D spatially resolved imaging spectrometer using relativistic pulse by Azamoum et al.^13^. **d** Future vision of high-dimensional relativistic spatiotemporal pulses for plasma study

The above method developed by the authors provides a way to observe the early stages of pre-plasma generation, which has not been previously studied in simulations or experiments. This observation is crucial for optimization of the subsequent ion acceleration during relativistic laser-thin foil interactions. Moreover, their approach contributes to the fundamental understanding of the evolution details of pre-plasma formation, which is essential to applications involving low- and high-power laser-matter interactions. In particular, for relativistic-induced transparency (RIT) acceleration scheme^15^, the characterization of the early stage formation from solid-to-overdense-plasma paves the way for improving the proton energy and advancing the practical application of laser-accelerated ion technologies. Furthermore, the developed simulation models can be used for thicker foils and thick nontransparent targets with relative modifications.

Looking forward, with the rapid development of ultrashort laser technology, new types of high-dimensional spatiotemporal ultrashort laser pulses may significantly impact research related to plasma. The high dimensionality, not only involves conventional laser parameters such as the laser contrast, but also couples spatiotemporal properties characterized by Hermite-Gaussian (HG) beam^16^, longitudinal or transverse orbital angular momentum (OAM)^17^, optical torques^18^ etc. With the complexity of the high-dimensional irradiating or probing laser pulses, the formation and observation of state from solid-to-overdense plasma remain open questions. Early works have shown astonishing results on the interactions of ultraintense vortices and plasmas, such as the hollow plasma acceleration^19^, proton acceleration with intense twisted laser light^20^, and interaction of ultraintense laser vortices with plasma mirrors^21^. The information embedded in the highly structured ultrashort laser pump pulses might be delivered to the plasmas and the generated high-power particles such as ions and electrons. Furthermore, high-dimensional spatiotemporal laser pulses can serve as effective probing methods^22,23^ to sense the transition process of the pre-plasma, providing insights beyond transmission measurement, such as phase information. The proposed simulation model by the author and its possible extension might be used to calculate the interaction of high-dimensional spatiotemporal laser pulses with targets, which is also crucial for understanding and describing the transition. This combination of high-dimensional ultrashort laser pulses and plasmas might propel the technique and the understanding of plasma dynamics to a new level. Overall, the proposed method by the authors shows the observation of solid-to-overdense-plasma transition over sub-picosecond timescales with single-shot probing for the first time, both in simulation and experiment. Future studies extending the method to high-dimensional ultrashort laser pulses may lead to a better understanding and more intricate manipulation of the plasma dynamics.
